# A Comprehensive Overview of the Cyclodipeptide Synthase Family Enriched with the Characterization of 32 New Enzymes

**DOI:** 10.3389/fmicb.2018.00046

**Published:** 2018-02-12

**Authors:** Muriel Gondry, Isabelle B. Jacques, Robert Thai, Morgan Babin, Nicolas Canu, Jérôme Seguin, Pascal Belin, Jean-Luc Pernodet, Mireille Moutiez

**Affiliations:** ^1^Institute for Integrative Biology of the Cell (I2BC), CEA, CNRS, Univ. Paris-Sud, Université Paris-Saclay, Gif-sur-Yvette, France; ^2^SIMOPRO, Institut Frédéric Joliot, CEA-Saclay, Gif-sur-Yvette, France

**Keywords:** secondary metabolites, biosynthetic pathways, cyclodipeptide synthase, tRNA-dependent enzymes, diketopiperazine, activity prediction, cyclodipeptide MS/MS

## Abstract

Cyclodipeptide synthases (CDPSs) use as substrates two amino acids activated as aminoacyl-tRNAs to synthesize cyclodipeptides in secondary metabolites biosynthetic pathways. Since the first description of a CDPS in 2002, the number of putative CDPSs in databases has increased exponentially, reaching around 800 in June 2017. They are likely to be involved in numerous biosynthetic pathways but the diversity of their products is still under-explored. Here, we describe the activity of 32 new CDPSs, bringing the number of experimentally characterized CDPSs to about 100. We detect 16 new cyclodipeptides, one of which containing an arginine which has never been observed previously. This brings to 75 the number of cyclodipeptides formed by CDPSs out of the possible 210 natural ones. We also identify several consensus sequences related to the synthesis of a specific cyclodipeptide, improving the predictive model of CDPS specificity. The improved prediction method enables to propose the main product synthesized for about 80% of the CDPS sequences available in databases and opens the way for the deciphering of CDPS-dependent pathways. Analysis of phylum distribution and predicted activity for all CDPSs identified in databases shows that the experimentally characterized set is representative of the whole family. Our work also demonstrates that some cyclodipeptides, precursors of diketopiperazines with interesting pharmacological properties and previously described as being synthesized by fungal non-ribosomal peptide synthetases, can also be produced by CDPSs in bacteria.

## Introduction

Cyclodipeptide synthases (CDPSs) are a novel family of enzymes that use aminoacyl-tRNAs (aa-tRNAs) as substrates for the biosynthesis of various cyclodipeptides (Gondry et al., 2009; Moutiez et al., 2017), precursors of diketopiperazines (DKPs), a large class of natural products with noteworthy biological activities (Borthwick, 2012). Since the first description of a CDPS enzyme in 2002 (Lautru et al., 2002), 66 members have been characterized for their cyclodipeptide-synthesizing activities (Gondry et al., 2009; Seguin et al., 2011; Giessen et al., 2013a,b; Alqahtani et al., 2015; Jacques et al., 2015; James et al., 2016; Brockmeyer and Li, 2017; Patteson et al., 2017). Most CDPSs are promiscuous enzymes, synthesizing one main cyclodipeptide and one or several minor cyclodipeptides. Globally, they synthesize about 55 different cyclodipeptides made up of 17 proteinogenic amino acids.

We recently showed that CDPSs divide into two phylogenetically distinct subfamilies named NYH and XYP, according to the identity of a trio of essential residues (Jacques et al., 2015). The NYH enzymes have been extensively characterized and the crystal structures of three of them, AlbC from *Streptomyces noursei*, Rv2275 from *Mycobacterium tuberculosis* and YvmC from *Bacillus licheniformis*, are available (Vetting et al., 2010; Bonnefond et al., 2011; Sauguet et al., 2011; Moutiez et al., 2014a). These CDPSs adopt a common architecture with a monomer containing a Rossmann-fold domain. The catalytic mechanism used by these three CDPSs has been investigated (Vetting et al., 2010; Bonnefond et al., 2011) and fully elucidated for AlbC (Sauguet et al., 2011; Moutiez et al., 2014a). The AlbC catalytic cycle begins with the binding of the first aa-tRNA, with its aminoacyl moiety accommodated in a surface-accessible pocket P1 and transferred onto a conserved serine residue to form an aminoacyl-enzyme intermediate. The second aa-tRNA interacts with this intermediate so that its aminoacyl moiety, accommodated in a wide cavity P2, is transferred to the aminoacyl-enzyme to form a dipeptidyl-enzyme intermediate. Finally, the dipeptidyl moiety undergoes an intramolecular cyclization leading to the final cyclodipeptide.

We previously proposed an approach to predict the main cyclodipeptide produced by yet-to-be characterized CDPSs (Jacques et al., 2015). Briefly, structural studies on the CDPS AlbC led to the identification of the two pockets P1 and P2 and showed that these pockets are bordered by eight and seven residues, respectively (Sauguet et al., 2011; Moutiez et al., 2014a). Assuming that the positions of residues lining P1 and P2 are conserved in all CDPSs and that their nature is related to the recognized aminoacyl moiety, specificity sequence motifs were defined for P1 and P2 for all putative new CDPSs. These motifs were used in combination with phylogenetic distribution to predict the main product of yet-to-be characterized CDPSs and to classify them into different specificity-based groups, according to the predicted product. For CDPSs producing the same main cyclodipeptide, the residues lining P1 and P2 appear well conserved within a subfamily, allowing the definition of consensus motifs specific to various specificity-based groups of CDPSs (Jacques et al., 2015). Six groups containing at least five characterized members were shown to be predictable with a good level of confidence (Jacques et al., 2015). For four of these groups, consensus motifs of P1 and P2 pockets have been identified and correspond to the synthesis of cWW, cLL, cCC, or cAE as the main cyclodipeptide. For the two others, the consensus motif is known for only one of the two pockets and correspond to the synthesis of a glutamyl- or alanyl-containing cyclodipeptide, respectively named cXE and cAX (X means that the nature of the other amino acid incorporated differs according to the CDPS considered). Few other groups could be postulated but not formally established as they contained only one to three characterized enzymes. On the 257 characterized and putative CDPSs identified in the National Center for Biotechnology Information (NCBI) database in 2015, 42% belonged to a well-defined group, 40% to other putative groups not formally defined and 18% had unpredictable activities. This last group is of particular interest since CDPSs with unpredictable activities are likely to produce new cyclodipeptides (Jacques et al., 2015).

Here, we describe 32 new active CDPSs, most of them chosen among the putative enzymes with unpredictable activities. This allows us to identify new cyclodipeptides produced by CDPSs and to define new consensus motifs, thus improving the predictive model of CDPS activity. We updated the list of putative CDPSs found in the NCBI database and retrieved about 500 new sequences (June 2017), which were analyzed with the improved predictive model to propose the main cyclodipeptide they produce. We also consider the phylum distribution of the family and potential relationship with CDPS specificities. Finally, we characterized several CDPSs synthesizing cyclodipeptides that were also previously shown to be synthesized by non-ribosomal peptide synthetases (NRPSs).

## Materials and methods

### Bioinformatics analyses

The BLAST tools and resources of NCBI databases have been routinely used. Sequences truncated at the N-terminus were corrected when possible. For each of these proteins, we examined the 5′ surrounding DNA sequence of the annotated gene to identify an alternative start codon located upstream, leading to an extended amino acid sequence that contains all catalytic residues and matches correctly with the N-terminal part of the previously characterized CDPSs. Sequences truncated at the C-terminus or lacking the catalytic serine were removed. Only one sequence was kept when several sequences exhibiting more than 98% sequence identity were found. Multiple sequence alignments were done using Muscle, integrated into Seaview (Gouy et al., 2010). Alignments were further manually curated for accurate alignment of catalytic residues. HHPred analyses were performed for sequences with catalytic residues variations, making not obvious the alignment of catalytic residues (Alva et al., 2016). Residues lining P1 and P2 were determined from these alignments, using data obtained with AlbC (Moutiez et al., 2014a). Eight residues line P1 (Residues 33-35-65-67-119-185-186-200, AlbC numbering) while seven residues line P2 (Residues 152-155-156-159-204-206-207, AlbC numbering). The phylogenetic trees were calculated using the PhyML program (v 3.1) based on maximum-likehood method (LG substitution model and NNI tree searching operation) (Lefort et al., 2017). The iTOL suite was used to generate graphical representation of phylogenetic trees (Letunic and Bork, 2016). Sequence consensus motifs were represented as logos, obtained at the WebLogo website (http://weblogo.berkeley.edu/; Schneider and Stephens, 1990; Crooks et al., 2004).

### CDPS genes

Synthetic genes encoding CDPSs 62-103 and optimized for expression in *E. coli* were obtained from GeneArt. They were designed on the same basis as in Jacques et al. (2015), i.e., they were designed to have an *Nco*I restriction site (*CCATGG*) containing the ATG start codon and a *Bgl*II restriction site located downstream from the last codon of the coding sequence. If the residue following the initiating methionine could not be encoded by a GXX codon, an alanine-encoding codon (GCA) was introduced after the start codon to complete the *NcoI* motif. The synthetic genes were provided in GeneArt specific cloning vectors. Their sequences are given in Supplemental Data Set 1. CDPS coding sequences were then inserted between the *Nco*I and *Bgl*II restriction sites of pIJ196 for protein expression in *E. coli* (Jacques et al., 2015). All recombinant plasmids were prepared using DH5α bacteria and verified by DNA sequencing of the promoter and CDPS-encoding regions (Eurofins-MWG).

### Expression of CDPSs and sample preparation

Each recombinant putative CDPS was expressed in *E. coli* BL21AI-pREP4 in medium throughput format from the corresponding pIJ196-derived plasmid (Jacques et al., 2015). An expression trial was performed in *E. coli* M15-pREP4 for CDPSs found inactive or poorly active in BL21AI-pREP4. Bacteria were cultured in 10 ml 24-well plates with round-bottomed wells (Whatman/GE Healthcare) containing 2 ml of the appropriate growth medium, covered with a hydrophobic porous film (VWR), and shaken at 200 rpm. Starter cultures were M9-derived minimum medium supplemented with trace elements and vitamins (Gondry et al., 2009), 200 μg/ml ampicillin, 25 μg/ml kanamycin and 0.5% glucose. They were inoculated with several colonies from competent bacteria freshly transformed with plasmids encoding CDPSs. After an overnight incubation at 37°C, the starter culture was used to inoculate (1/50) the same M9-derived minimum medium except that glucose was replaced by a combination of 0.5% glycerol, 0.05% glucose, and 0.02% lactose for BL21AI-pREP4 bacteria (Studier, 2005) or by 0.5% glycerol for M15-pREP4 bacteria. M15-pREP4 bacteria were grown at 37°C until the OD_600_ reached 0.6, and expression of the putative CDPS was induced by the addition of isopropyl-β-D-thiogalactopyranoside (IPTG, 2 mM final concentration). Cultivation was continued for 24 h at 20°C. BL21AI-pREP4 bacteria were grown in an auto-induced medium (Studier, 2005) and thus did not need the addition of IPTG for CDPS expression. After inoculation of the expression cultures, BL21AI-pREP4 bacteria were grown at 37°C for 3.5 h, and transferred to 20°C for 20.5 h. At the end of cultivation, cells were pelleted by centrifugation of the plates. The supernatants were collected, acidified (2% TFA final concentration) and frozen at −20°C.

### Cyclodipeptide identification

Cyclodipeptides were detected by LC-MS/MS analyses on an Agilent 1100 HPLC coupled via a split system to an Esquire HCT ion trap mass spectrometer (Bruker Daltonik GmbH) set in positive mode. Samples were loaded onto an Altantis dC18 column (4.6 × 150 mm, 3 μm, 100 Å, Waters) or on a Hypercarb column (4.6 × 150 mm, 5 μm, 250 Å, ThermoScientific), developed over 50 min with the linear gradient 0–50% (v/v) (solvent A: 0.1% (v/v) formic acid in H_2_O, solvent B: 0.1% (v/v) formic acid in acetonitrile/H_2_O (90/10), flow rate, 0.6 ml/min). Analysis on dC18 column proved to be efficient to detect most of the cyclodipeptides, except the most polar ones such as cGG, cGN (Jacques et al., 2015). Hypercarb column efficiently separates hydrophilic cyclodipeptides but is not suitable for the separation of aromatic-containing ones. Positive electrospray ionization and mass analysis were optimized for the detection of compounds in the range of natural cyclodipeptides. For MS/MS, an isolation width of 1.0 m/z was set for isolating the parent ion, and a fragmentation energy ramp was used for optimizing the MS/MS fragmentation process. All data were acquired and processed using software from the manufacturer (Bruker Daltonik GmbH).

Cyclodipeptides were detected and identified from both the m/z value of their [M+H]^+^ species (MS) and their daughter ion spectra (MS/MS), as a result of their common fragmentation patterns. The identification of the detected cyclodipeptides was done from data gathered in Table S1 and Supplemental Data Set 2. Table S1 has been established from a compilation of our experimental results on authentic standards (bought or chemically synthesized in the lab, see Supplemental Data Set 2), completed by relevant literature data (Papayannopoulos, 1995; Chen et al., 2004; Stark and Hofmann, 2005; Xing et al., 2008; Guo et al., 2009; Jacques et al., 2015).

The identity of cPR, also known as verpacamide A, was confirmed by comparison with an authentic standard (Vergne et al., 2006), which was also used to obtain a calibration curve relating mass concentration and peak area at 214 nm.

Cyclodipeptides were quantified on the basis of their peak area at 214 nm, possibly converted in mg/L of culture using calibration curves performed with standards, when commercially available (see Supplemental Data Set 2).

## Results

### Newly characterized CDPSs and the cyclodipeptides they produce

We investigated the production of cyclodipeptides of 42 selected sequences (designated CDPSs 62-103) (Supplemental Data Set 1). Thirty sequences were selected from the 257-sequence set published in 2015 (Jacques et al., 2015) and 12 from a new set obtained from analyses performed in March 2016. Thirty-six of the 42 selected enzymes cannot be associated with any obvious predictable activity using the model proposed by Jacques et al. Nine are XYP members and 27 are NYH members. The discrepancy between the number of XYP and NYH CDPSs selected is directly related to the much smaller number of XYP sequences available in databases. The six remaining proteins were chosen to assess the predictive value of several putative specificity-groups. Four are NYH (CDPSs 94-97) and two are XYP proteins (CDPSs 85-86), which may belong to putative specificity-groups that synthesize cYY and cLI/cLL, respectively (Jacques et al., 2015). Among the 27 unpredictable NYH members selected, ten (CDPSs 62-66, 68-71, 103) had been put into the putative specificity-group containing AlbC by Jacques et al., suggesting that the main cyclodipeptide they synthesize is a phenylalanyl-containing cyclodipeptide, named cFX (with X differing depending on the CDPS considered) (Jacques et al., 2015). However, this grouping has been brought into question by the recent characterization of one member, NozA, as a cWW-synthesizing enzyme, whereas it was grouped with AlbC (Alqahtani et al., 2015; James et al., 2016). Characterization of this subset thus aimed to identify the compounds formed by members of this group, formerly thought to be homogenous. We cloned each of the genes in a vector allowing their expression in *E. coli*. We used the medium-throughput method previously described to recover the cyclodipeptides synthesized by CDPSs in culture supernatants (Jacques et al., 2015). These supernatants were then analyzed by LC-MS/MS to detect and identify the cyclodipeptides produced.

**Figure 1:**
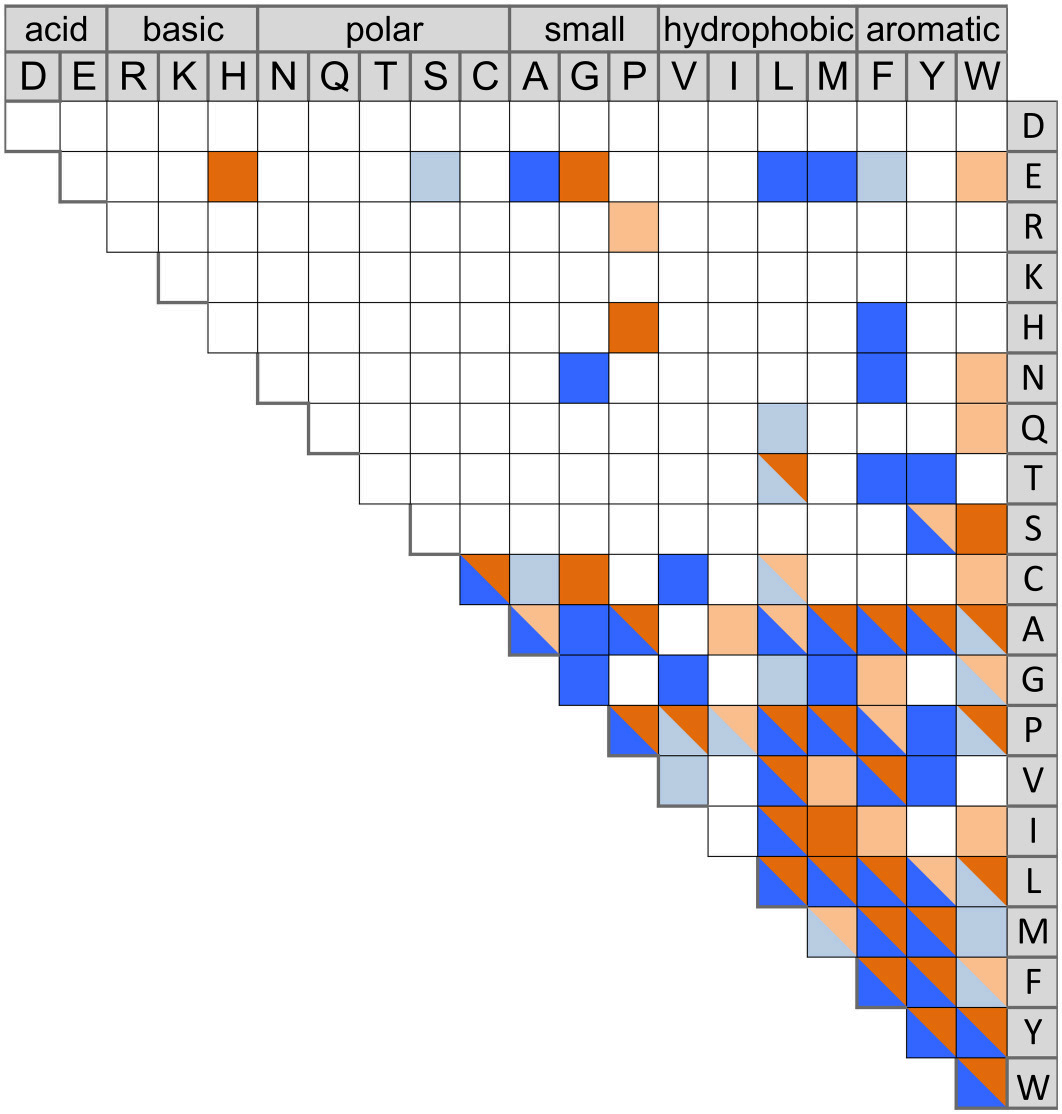
Current diversity of the cyclodipeptides produced by the CDPS family. The amino acids forming the cyclodipeptide ring are indicated in the one-letter code at the top and right. Previously identified cyclodipeptides are in blue, cyclodipeptides identified with the present set of CDPSs are in orange. Light colors indicate cyclodipeptides detected in low amounts (UV peak area at 214 nm less than 200). The distribution of cyclodipeptides according to NYH or XYP subfamilies is shown in Figure S1.

Thirty-two of the 42 selected CDPSs had cyclodipeptide-synthesizing activity under the conditions tested (Table 1; Supplemental Data Set 3). There is no obvious explanation for the absence of activity of the others (see the end of the 4th paragraph of the Results section). Altogether, the 32 active CDPSs produced 52 cyclodipeptides, among which 16 have not been previously observed as CDPS products (Figure 1). In addition, five cyclodipeptides previously detected only at trace levels (Jacques et al., 2015) were now obtained in significant amounts (Figure 1). We observed for the first time, the incorporation of a basic residue into a CDPS product, as NYH CDPS 83 synthesizes cPR (Table 1). This brings the number of amino acids now incorporated into cyclodipeptides to 18 of the 20 proteinogenic ones (except for D and K), and the total number of cyclodipeptides synthesized to date by CDPSs to 75 of the 210 natural cyclodipeptides made up of proteinogenic amino acids.

**Table 1:** Cyclodipeptides produced by the newly characterized CDPSs.

**CDPS**	**Species**	**CDPS subfamily**	***In vivo* activity (BL21 AI-pREP4)^a^**	**UV_214nm_ area of main compound**	**Quantity in mg/L culture^b^**
62	*Nocardiopsis potens*	**NYH**	**cFM** (30%), **cFF** (30%), cFA (19%), cFY (8,4%), cFL (7%), *cYA, cFW, cMM, FV, cLM, cLY*	2,650	cFM 8.8, cFF 7
63	*Streptomyces lavendulae*	**NYH**	**cWW** (62%), cWP (15,8%), cWA (9,8%), cWL (6,2%), *cWS (2%), cWQ, cWE, cWF,cWC, cWG, cWI, cWN*	14,802	cWW 20
64	*Streptomyces purpureus*	**NYH**	**cWW** (100%)	9,573	cWW 12.7
65	*Nocardiopsis xinjiangensis*	**NYH**	–		
66	*Actinomadura oligospora*	**NYH**	**cFY** (94,3%), cYY (5,7%)	2,729	cFY 6.5
67	*Streptomyces catenulae*	**NYH**	**cFY** (99,9%)	1,969	cFY 5.1
68	*Streptomyces* sp. *NRRL F-5053*	**NYH**	**cLW** (97%), *cLL, cFW, cFL*	11,431	cLW 13.6
69	*Streptomyces rimosus*	**NYH**	**cWY** (98%), cWW (2%)	11,709	cWY 7
70	*Streptomyces* sp. *NRRL B-24484*	**NYH**	**cWW** (73,2%), cWL (16,5%), cWS (8,9%), *cWQ, cWF, cWN, cWA,cWP, cWE, cWG*	18,457	cWW 39
71	*Strepto. roseochromogenes subsp. Oscitans DS 12.976*	**NYH**	**cWY** (98%), cWA (2%), *cWS*	4,667	cWY 3
72	*Streptomyces* sp. *AW19M42*	**NYH**	–		
73	*Streptomyces aureocirculatus*	**NYH**	cWA (60%), cWP (40%)	138	
74	*Streptomyces* sp. *NRRL S-1868*	**NYH**	**cWP** (99,5%), *cWA, cWS*	15,655	
75	*Streptomyces* sp. *NRRL F-5123*	**NYH**	**cWP** (99,5%), *cWF, cWG*	7,616	
76	*Streptacidiphilus albus*	**NYH**	–		
77	*Streptomyces scabrisporus*	**NYH**	**cLV** (56,2%), cLT (5,9%), **cLL** (18%), cLI (18,3%), *cLA*	1,715	cLV 5.5, cLL 6, cLI 6
78	*Kibdelosporangium aridum*	**NYH**	**cFG** (100%)	51	cFG < 0.1
79	*Kibdelosporangium aridum*	**NYH**	–		
80	*Streptomyces natalensis*	**NYH**	–		
81	*Streptomyces roseoverticilatus*	**NYH**	–		
82	*Streptomyces varsoviensis*	**NYH**	**cPL** (65,8%), cPA (20%), cPV (7,6%), *cPF, cPM, cLL, cLA, cPI, cAA*	5,945	cLP 2
83	*Lysobacter antibioticus*	**NYH**	**cPR** (100%)	140	cPR 4
84	*Allokutzneria albata*	**NYH**	**cFI** (100%), *cLI, cMI*	54	cFL 0.2
85	*Actineokineospora* sp. *EG49*	**XYP**	**cLI** (65,7%), cMI (9,1%), cLL (5,4%), cML (3,3%), cLV (4,9%), cLP (4,6%), *cLA (3,3%), cLC, cMV, cMP, cMA, cIA*	4,220	cLI^c^ 46, cLL 3.7
86	*Hahella ganghwensis*	**XYP**	**cLL** (50,6%), cLI (17,9%), cLP (10,6%), cLA (9,1%), cLM (7%), cLV (4,2%), *cLC, cMI, cLT, cMA, cMP, cLY, cMV*	926	cLL 10, cLI^c^ 3.5
87	*Legionella lansingensis*	**XYP**	**cGE** (100%)	3,121	cGE 14
88	*Geminicoccus roseus*	**XYP**	–		
89	*Algicola sagamiensis*	**XYP**	**cAP** (97%), cAA (3%)	3,454	
90	*Parcubacteria bacterium RAAC4_OD1_1*	**XYP**	cHP (44%), cHE (56%)	688	cHP 0.4
91	*Methylovulum miyakonense*	**XYP**	**cFF** (51,5%), cFY (36,1%), cFL (8,2%), cFM (3,5%), *cYM*	2,786	cFF 6.5, cFY 4, cFL 1.4
92	*Aminiphilus circumscriptus*	**XYP**	–		
93	*Fusarium fujikuroi IMI 58289*	**XYP**	–		
94	*Streptomyces katrae*	**NYH**	**cYY** (71,3%), cYF (14,9%), cYA (10,7%), cYM (2%), *cFF, cFA, cYL, cYS*	12,312	cYY 29.3, cYF 6.6, cYA 5.5
95	*Streptacidiphilus melanogenes*	**NYH**	**cYY** (59,6%), cYA (22,2%), cYF (16,3%), *cYM, cFF, cFA, cYL , cYS*	11,078	cYY 26.4, cYA 12.3, cYF 8
96	*Streptomyces* sp. *PCS3-D2*	**NYH**	**cYY** (57,7%), cYA (22,2%), cYF (17,5%), *cYM, cFF, cFA, cYL*	10,901	cYY 26, cYA 12.5, cYF 8.6
97	*Streptomyces peruviensis*	**NYH**	**cYY** (58,4%), cYA (28,8%), cYF (11,2%), *cYM, cFF, cFA, cYL*	10,095	cYY 24, cYA 14.8, cYF 5
98	*Vibrio sagamiensis*	**NYH**	**cPM** (95,4%), cMA (4,6%), *cPA*	6,265	cPM 2.6
99	*Lyngbya confervoides*	**XYP**	–		
100	*Nocardia concava*	**NYH**	**cPP** (100%)	1,871	
101	*Thalassomonas viridans*	**XYP**	**cYY** (98%), cYM (2%)	3,406	cYY 8.1, cYM 0.2
102	*Algicola sagamiensis*	**NYH**	**cCG** (72,8%), cCC (27,2%)	596	
103	*Streptomyces* sp. *HPH0547*	**NYH**	**cFL** (91%), cFM (7%), *cFF, cYL, cLM*	16,190	cFL 21.3, cFM 1.6

a *Cyclodipeptides produced by BL21 AI-pREP4 expressing the recombinant CDPS; cyclodipeptides are ranked according to the peak area on UV chromatograms recorded at 214 nm (see also Supplemental Data Set 3). Percentages in brackets indicate the proportion of each cyclodipeptides and were calculated from these UV peak areas. The most abundant cyclodipeptide produced is in bold. Cyclodipeptides representing less than 2% are in italic*.

b *Quantities of cyclodipeptide produced are given in gray when standard are available for establishing of calibration curves (see also Supplemental Data Set 2)*.

c *cLI quantities were estimated using the calibration curve for cLL*.

The newly characterized NYH CDPSs synthesize 22 additional cyclodipeptides, completing the set of products obtained with the former characterized NYH group, bringing this set to 52 cyclodipeptides (Figure S1A). The NYH enzymes now incorporate 17 of the 20 proteinogenic amino acids, except for H, D, and K. The newly characterized XYP CDPSs produced nine additional products. This new set of XYP enzymes produced a total of 51 different cyclodipeptides, in which are incorporated 17 of the 20 proteinogenic amino acids, differing from NYH members by the exclusion of R instead of H (Figure S1B).

### The contribution of the newly characterized CDPSs to the determination of specificity groups

We examined the main cyclodipeptide synthesized by each of the newly characterized CDPSs, together with the sequence motifs of the binding pockets and the phylogenetic proximity of the considered enzymes (Table 2).

**Table 2:** Sequence motifs of the pockets P1 and P2 of characterized CDPSs, colored according to the aminoacyl mainly accommodated^*^ (inferred from sequence alignments and proximity with characterized CDPSs or enzymes synthesizing homo-cyclodipeptides).

	**Pocket P1**		**Pocket P2**	
**CDPS**	**Amino acyl mainly accommodated**	**Sequence motifs**	**Amino acyl mainly accommodated**	**Sequence motifs**
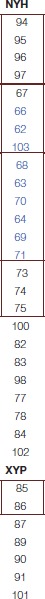
	Y	VGITMLFN	Y	LRFDQLP
	Y	VGVTMLFN	Y	LAYSQLP
	Y	VGITMLFN	Y	LAFSQLP
	Y	VGVTMLFN	Y	LSFEQLP
	F	VGITMFFV	Y	MHFGVTP
	F	LGVAIFLC	Y	HFVGKLP
	F	LGVVLFFT	F/M	QDFDKMP
	F	LGLVLFLT	L	QALEKMP
	W	LGVALFLS	L	MSFGMAQ
	W	LGVALFFA	W	MLHGVMP
	W	LGVALFFA	W	MGFAEPL
	W	LGVALFFH	W	MQFKTLA
	W	LGIALFFS	Y	MLHGLTP
	W	LGVALFFS	Y	MTFPQIP
	W	LGIPLFFV	P/A	VRARAGF
	W	LGIPLFFV	P	ARAVMVP
	W	LGVPLLLA	P	ASVGKID
	P	CGHPWLLY	P	TDVHRIP
	P	LGFPWFMF	L	LKAGQFE
	P	VGLPWFFF	R	ATAGRIS
	P	LGLPWLFF	M	NQCNSFR
	L	LGFPWLVF	V/L/I	TGARRMD
	F/G ?	LGLAWIGC	F/G ?	VAYHRLA
	F/I ?	LGLPWFLF	F/I ?	IRVTRSP
	C	VGYPSIFF	G/C	SAIKDPT
	L/I ?	FGLGCVFM	L/I ?	FRYRGFS
	L/I ?	FGLGCIFF	L/I ?	FSYRGFG
	G	LFVAWIVI	E	NTYRKLT
	A/P ?	FAYVHAVN	A/P ?	FFYRGID
	H	GAYAWELY	P/E	FEWKYTR
	F	FGLAWSLL	F/Y	LNYSTYA
	Y	FPLCWISE	Y	LKLRREE

* *Orange: Y, blue: F, green: W, yellow: P, violet: L. When accommodation in P1 or P2 could not be deduced from existing data, amino acyl accommodated are indicated in both pockets and signaled by a question mark. Groups of CDPSs phylogenetically close are framed. NYH CDPSs previously grouped in the same specificity group than AlbC are labeled in blue*.

CDPSs that synthesize the same main product but belong to either the XYP or NYH subfamily (e.g., NYH CDPSs 94-97 and XYP CDPS 101, which synthesize cYY), have pockets with different sequence motifs. This feature had already been observed for cLL-synthesizing enzymes (Jacques et al., 2015) and appears to be the general case.

A few sets of CDPSs that synthesize the same main product share common features. The first set corresponds to NYH CDPSs 94-97, which synthesize cYY. The sequence motifs for P1 are highly conserved, with the only variable position occurring on the third residue (position 65, AlbC numbering), which is either valine or isoleucine. Four of the seven residues of pocket P2 are strictly conserved and a fifth residue is either phenylalanine or tyrosine. The combination of these two motifs is specific for this set of CDPSs. Other NYH CDPSs that bind a tyrosinyl in the second pocket, but synthesize cyclodipeptides other than cYY, exhibit different P2 motifs (e.g., CDPSs 66, 67, 69, and 71). The second set includes XYP CDPSs 85 and 86, which synthesize cLI or cLL as the main products and have highly similar P1 and P2 sequences motifs.

**Figure 2:**
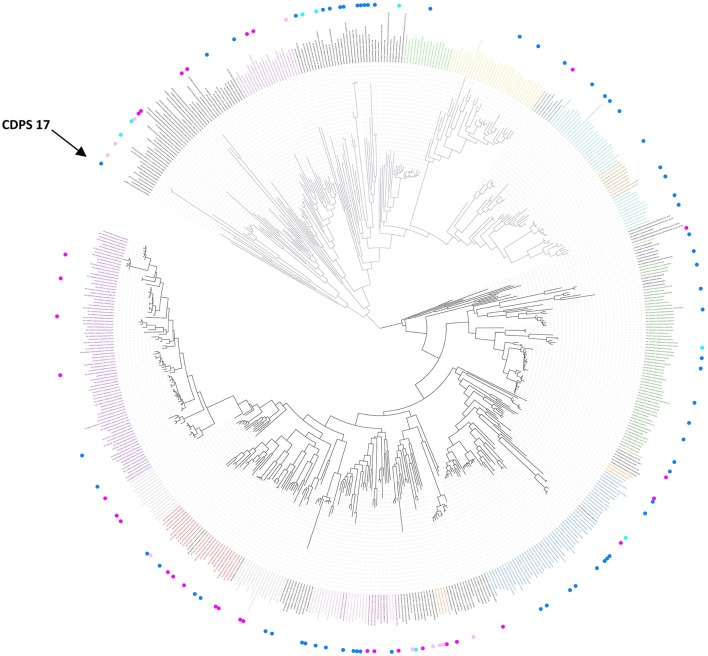
Phylogenetic tree of known and putative CDPSs (June 2017) (see also Supplemental Data Set 6 for a better resolution and aLRT values). The CDPSs experimentally characterized prior to this study are labeled with blue dots and CDPS characterized within this study with pink dots (dark for active ones, light for inactive ones). Branches corresponding to NYH CDPSs are black; those corresponding to XYP ones are gray. Labels of groups with predicted activity are colored respectively in green (cLL), blue (cCC), light pink (cWW), orange (cPX), dark pink (cW_1_X), gray (cW_2_X), red (cFX), violet (cYY) for NYH CDPSs, and light blue (cAE), brown (cLE), dark blue (cGE), yellow (cAA), light green (cGG), and fushia (cLI/cLL) for XYP CDPSs. The arrow indicates the CDPS 17, previously suspected to belong to a third CDPS subfamily (SYQ). See also Supplemental Data Set 5 for the sequence alignment of the whole set of CDPSs.

Nine of the 10 CDPSs previously grouped with AlbC were active (Table 1). Experimentally, they divided into two groups, synthesizing as the main product either a phenylalanine-containing cyclodipeptide (CDPSs 62, 66, 103) like AlbC or cWW (CDPSs 63, 64, 68-71) like NozA (James et al., 2016). As we previously demonstrated that phenylalanyl is accommodated in P1 for AlbC (Moutiez et al., 2014b), we can assume that CDPSs, that belong to the group of AlbC, also accommodate phenylalanyl in P1 whereas CDPSs of the second group accommodate tryptophanyl. Sequence motifs of P1 must have sufficient differences to allow the accommodation of one or the other residue. Examination of P1 motifs (Table 2) showed that CDPSs accommodating tryptophanyl always have the pair VA or IA at positions 3 and 4 of P1, whereas CDPSs that accommodate phenylalanyl have different combinations of amino acids. However, one of the enzymes that preferentially accommodates phenylalanyl has the VA pair (CDPS 66), showing that discrimination between phenylalanyl and tryptophanyl by P1 is complex and probably involves other not yet identified specific residues. Both groups are nevertheless on separate branches of the phylogenetic tree (Table 2 and Figure 2).

CDPSs 73-75 mainly synthesize a tryptophanyl-containing cyclodipeptide but are not located in the same region of the phylogenetic tree as the tryptophanyl-accommodating CDPSs similar to NozA, described above. The only significant difference between these two CDPS sets occurs at the fourth residues of P1 (position 119, AlbC numbering), which is proline in the set containing CDPSs 73-75 and alanine in the other (CDPSs 63, 64, 68-71). Analysis of the amino acids lining the pocket P2 in these CDPSs did not reveal consensus motifs related to the recognition of a particular aminoacyl.

The last set contains CDPSs that synthesize a prolyl-containing cyclodipeptide as the main product (CDPSs 82, 83, 98, and 100). These enzymes are not closely related from a phylogenetic point of view (Figure 2) and their main product differs. Their P2 sequence motifs do not contain any common amino acids, but their P1 sequence motifs show some common features, suggesting that prolyl is bound by P1. However, other specificity determinants are likely involved in the recognition of prolyl because CDPSs with similar P1 motifs exhibit different specificities (e.g., CDPS 84, which mainly synthesizes cFI or CDPS 77, which synthesizes leucyl-containing cyclodipeptides). Studies of larger sets of CDPSs with the same specificity should provide further keys to connect essential sequence elements with the observed specificity.

### An enlarged set of predictable activities

We extended our analysis to all CDPSs biochemically characterized to date. This set now consists of 98 active CDPSs (Gondry et al., 2009; Seguin et al., 2011; Giessen et al., 2013a,b; Alqahtani et al., 2015; Jacques et al., 2015; James et al., 2016; Brockmeyer and Li, 2017; Patteson et al., 2017). The products and P1 and P2 motifs of all the enzymes previously characterized are shown in blue in Supplemental Data Set 4. CDPSs are grouped according to their subfamily, NYH or XYP, the main cyclodipeptide formed, and their phylogenetic proximity. Several sets now contain a sufficient number of members to implement the predictive method proposed by Jacques et al. (2015).

The clearest new predictable activity concerns the synthesis of cYY by NYH CDPSs related to Rv2275 (Gondry et al., 2009; Vetting et al., 2010). This group now encompasses six characterized enzymes, four of which (CDPSs 94-97) are from this study. These enzymes all possess similar motifs for P1 and P2, making it possible to define the consensus motifs reported in Table 3.

**Table 3:** Specificity groups identified and their consensus motifs of P1 and P2 determined from characterized CDPSs.

Specificity group (^*^)	**Consensus motif for P1**	**Consensus motif for P2**
**cWW (NYH)** (9 / 22)	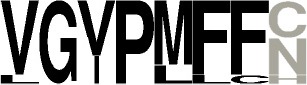	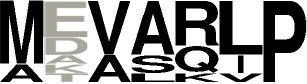
**cLL (NYH)** (10 / 123)	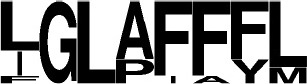	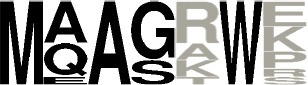
**cCC (NYH)** (11 / 60)	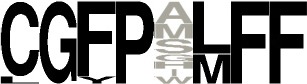	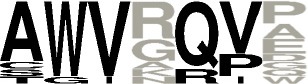
**cAE (XYP)** (8 / 55)	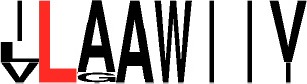	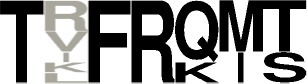
**cYY (NYH)** (6 / 145)	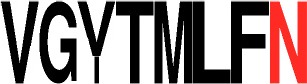	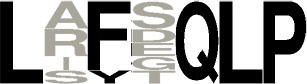
**cFX (NYH)** (8 / 31)	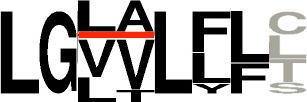	
**cW**_2_**X (NYH)** (7 / 34)	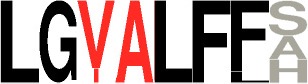	
**cPX (NYH)** (5 / 9)	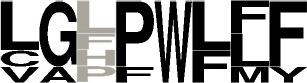	
**cLE (XYP)** (2 / 19)	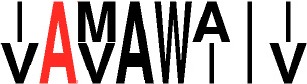	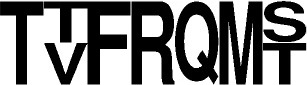
**cGE (XYP)** (1 / 7)	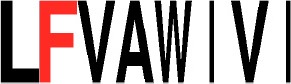	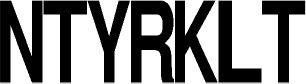
**cAA (XYP)** (2 / 33)	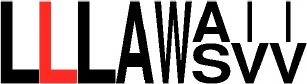	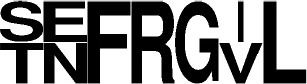
**cGG (XYP)** (1 / 17)	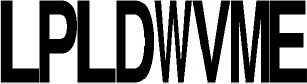	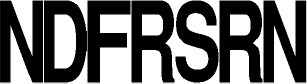
**cLI/cLL (XYP)** (4 / 34)	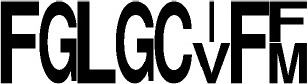	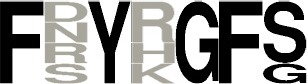
**cCX (NYH)** (12 / 61)	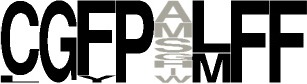	
**cW**_1_**X (NYH)** (12 / 36)	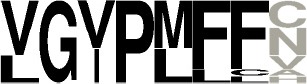	
**cXE (XYP)^a^** (9 / 81)		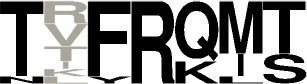
**cAX (XYP)^a^** (10 / 88)	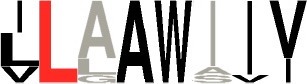	

Specificity groups are named according to the main cyclodipeptide synthesized; the family NYH or XYP of the CDPSs forming the group is indicated. The numbers in brackets (^*^) correspond to the number of CDPSs experimentally characterized vs. the total number of CDPSs in the group (Last up-date: June 2017). Groups for which less than five CDPSs have been experimentally characterized are labeled in blue. Sequence motifs correspond to residues suspected to delineate P1 and P2, determined for each CDPS (see Supplemental Data Set 4 and Table S2). They are presented as logo, corresponding to the frequency plot of amino acid at the different positions of P1 and P2 for characterized enzymes of the specificity group (see also Table S2 for comparison with logos obtained with enzymes of the whole specificity group). Degenerated positions are indicated in gray, amino acids likely to be essential for specificity are indicated in red.

a *The consensus motif for cXE was obtained using cAE, cLE and cGE groups of CDPSs, that for cAX was obtained using cAE and cAA groups*.

The other predictable groups are restricted to the prediction of the aminoacyl recognized by the first pocket P1, as key determinants for the specificity of P2 are not yet clear.

Two NYH groups are predicted to mainly synthesize a cWX. The first group (Table 3, group cW_1_X) contains the CDPSs 73-75, characterized in this study, together with the entire previously identified group that mainly synthesizes cWW (Jacques et al., 2015). The other group (cW_2_X) includes CDPSs 63, 64, 68-71, and NozA (James et al., 2016).

NYH CDPS 102 mainly synthesizes cCG and can be grouped with the group that mainly synthesize cCC, defining a cCX-synthesizing group (with X differing according to the CDPS).

Another group mainly synthesizes a cFX compound, as AlbC; it contains CDPSs 62, 66, 67, four previously characterized members (Gondry et al., 2009; Giessen et al., 2013b; Li et al., 2014; Jacques et al., 2015) and a recently identified member that produces mainly cFY (Brockmeyer and Li, 2017).

As noted above, predicting the synthesis of a cPX compound is challenging, as the characterized members are not phylogenetically grouped. We observed that the previously characterized CDPS 10 (Jacques et al., 2015), which mainly synthesizes mainly cPM, also has a sequence motif for P1 homologous to that of cPX-synthesizing CDPSs of this study. However, the prediction is uncertain, except for new CDPSs phylogenetically close to those already characterized.

Among the new XYP CDPSs, CDPS 87 exhibits a P2 sequence motif similar to that of the subgroup that synthesizes cXE cyclodipeptides (Jacques et al., 2015). Before our study, no clues were available concerning the specificity of the P1 pocket. We observed that CDPS 87 specifically produces cGE. This enlarges the number of XYP CDPSs considered to predict the binding of glutamyl in P2 and slightly modifies the previously proposed consensus sequence (Jacques et al., 2015).

### Overview of the updated family of CDPSs (June 2017)

A new search of the NCBI database in June 2017 retrieved 765 putative CDPSs after curating the initial set of hits. We constructed a phylogenetic tree with 568 CDPSs representative of the entire set: only one enzyme sequence was retained for CDPS sequences sharing more than 98% identical residues (Figure 2 and Supplemental Data Sets 5, 6). All putative CDPSs fall into the two previously identified subfamilies XYP and NYH. Previously characterized CDPS 17 (Jacques et al., 2015), suspected to be a representative of a third family (SYQ), now clearly appears to be a member of the XYP subfamily. NYH CDPSs form a larger group than their XYP homologs (507 vs. 258), but this persistent trend may be biased by the choice of genome sequencing projects. Experimentally characterized CDPSs are evenly distributed on the phylogenetic tree.

**Figure 3:**
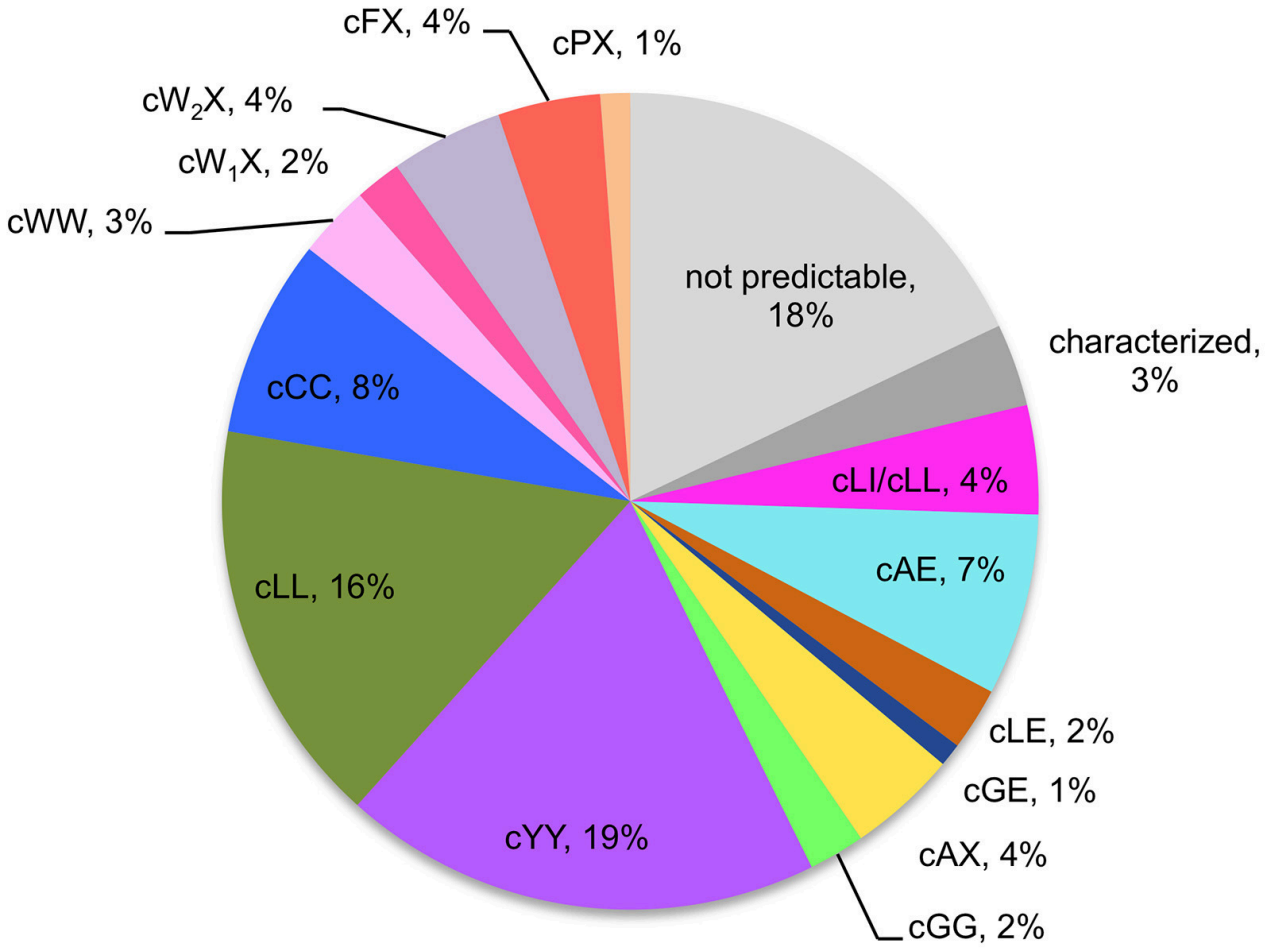
Predicted activities of the 765-CDPS set. The different specificities predicted are colored using the same code as for the phylogenetic tree (Figure 2). “Characterized” refers to enzymes experimentally characterized but not belonging to a predictable group.

CDPSs close in the phylogenetic tree and with patterns of P1 or/and P2 similar to those of characterized CDPSs were grouped together (Supplemental Data Set 4). In addition to the formally established specificity groups (This study; Jacques et al., 2015), we also considered five groups likely to synthesize cLI/cLL, cLE, cGE, cAA, and cGG as their main products. The number of CDPSs experimentally characterized for these groups, was between one and four. Table 3 shows the sequence motifs determined for P1 and P2 from characterized enzymes for all defined specificity groups. These groups appear in color in Figure 2 and their distribution by predicted activity is shown in Figure 3. Although the number of putative CDPSs has tripled since the last update (September 2014), the number of CDPSs with unpredictable activity is still approximately 18%. Sixty-six percent of the CDPSs are predicted to mainly synthesize one of the 10 cyclodipeptides for which the two amino acids can be predicted, i.e., cYY, cLL, cCC, and cWW for NYH CDPSs and cAE, cLI/cLL, cAA, cLE, cGG, and cGE for XYP CDPSs. Cyclodipeptides for which only one amino acid can be predicted (i.e., cWX, cFX, or cPX) are much more diverse (at least more than 12 different main products according to our experimental results) and are the main product of a restricted number of enzymes (11%). The number of XYP enzymes is higher than NYH enzymes (94 vs. 68) if CDPSs with unpredictable activity are considered (see Supplemental Data Set 4). Altogether, approximately 91% of the activity of NYH CDPSs can be predicted using their pocket sequence residues, whereas this figure falls to 73% for XYP CDPSs. Most of the CDPSs predicted to synthesize the same main cyclodipeptide, are on a same branch of the phylogenetic tree and form a distinct phylogenetic group.

The enzymes that were inactive under our experimental conditions fell into one main group in each subfamily. Most of the inactive enzymes for NYH CDPSs are clustered on the same branch and belong to actinobacteria. The observed inactivity may be due to errors in determining the start codon of the CDPS genes. New NCBI entries are now available for some of the enzymes studied and propose alternative start codons. Many inactive enzymes of the XYP CDPSs belong to fungi, cyanobacteria, or metagenomic species. The observed inactivity may be due to the set of *E. coli* tRNAs being unsuitable for a productive interaction with these enzymes, in addition to potential errors in determining the start codons.

### Phylum distribution and diversity of CDPSs

**Figure 4:**
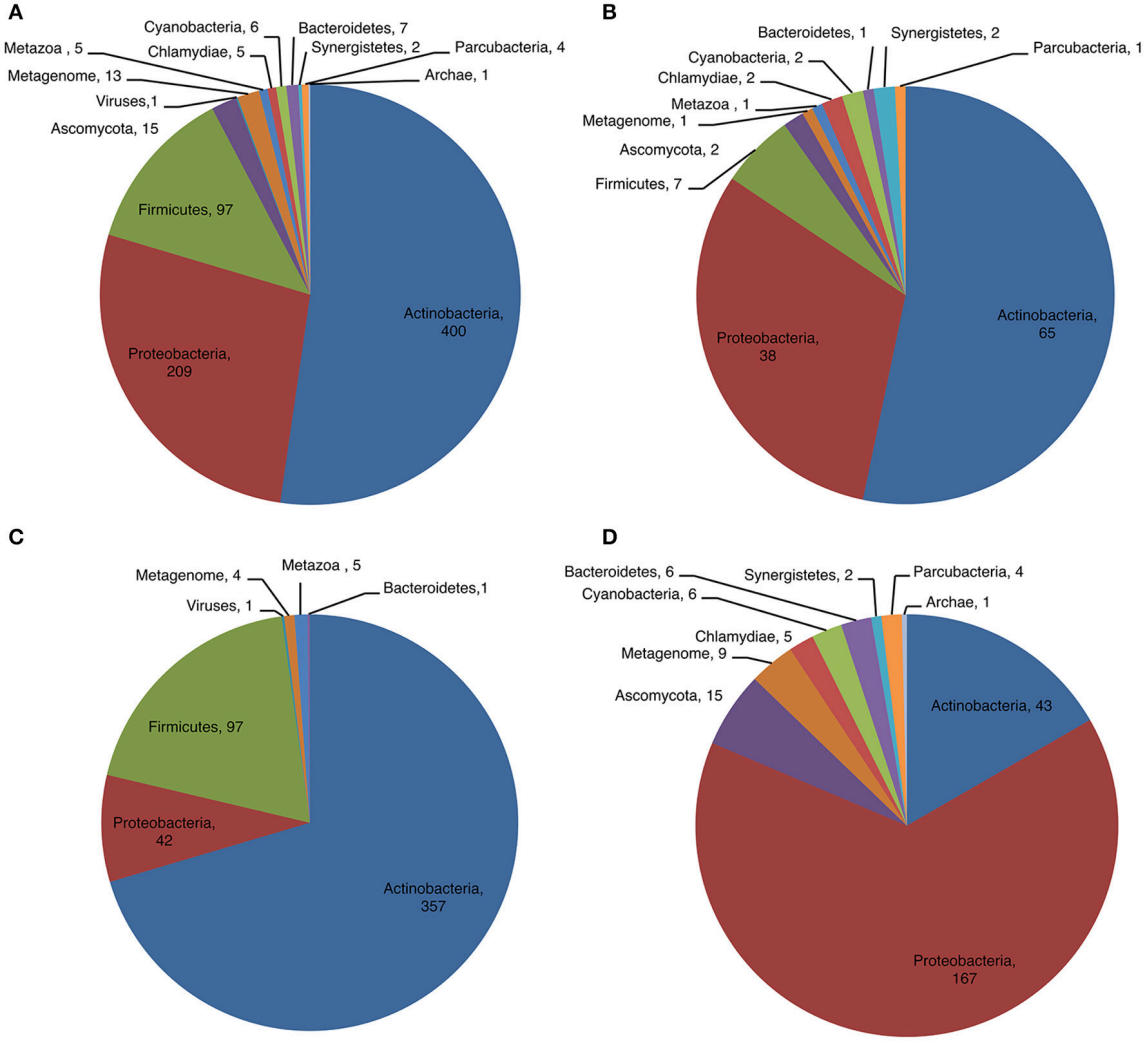
Phyla distribution of putative **(A)** and characterized **(B)** CDPSs and according to their belonging to NYH **(C)** or XYP **(D)** family.

We examined the distribution of CDPSs in terms of the organisms in which they are present (Figure 4). Experimentally characterized CDPSs are representative of the whole set (Figures 4A,B). Putative CDPSs appear in all domains of life. For the first time we retrieved a sequence annotated as originating from a metagenomic archaeon (AQS34941), that possesses all characteristics of a XYP CDPS. It has the greatest similarity with CDPS sequences from metagenomes, for which the specificity is unknown. The number of putative eukaryotic CDPSs has significantly increased (by a factor of approximately two relative to the set published in 2015). Eukaryotic CDPSs within the XYP subfamily originate from fungi. Two attempts to express a fungal CDPS have been made in *E. coli* and were unsuccessful (This study; Jacques et al., 2015). The identity of their potential product(s), if any, remains to be determined in a more appropriate assay. The four newly identified eukaryotic CDPS sequences of the NYH subfamily originate from marine species (coral, sea anemones or marine worms), like the already characterized Nvec-CDPS2 (Seguin et al., 2011). The distribution of phyla within NYH and XYP subfamilies is very different (Figures 4C,D). Most NYH CDPSs originate from actinobacteria (71.8%); Firmicutes (19.1%), with all CDPSs synthesizing or predicted to synthesize mainly cLL; and Proteobacteria (8.5%). Most NYH CDPSs are similar in length, but some enzymes are fused to a second protein domain in the C-terminal part; putative methyltransferase, P450 or ABC-transporter domains are found. NYH CDPS 71 is thus part of a larger protein consisting of two domains: an N-terminal CDPS moiety of approximately 240 amino acids fused to a 410 amino-acid C-terminal P450 domain. We found the isolated N-terminal domain to synthesize significant quantities of cWY. This result suggests that CDPSs can be functional within larger multi-domain proteins containing tailoring domains. Most XYP CDPSs (Figure 4D) come from proteobacteria (65.1%), followed by actinobacteria (16.7%). The remaining 18% are distributed among eight different phyla. The much larger diversity in sequence features observed in XYP CDPSs relative to those of the NYH subfamily may be related to this diverse phyla in which they are found. Indeed, some XYP CDPSs contain large amino acid insertions or deletions relative to the structurally characterized enzymes (Jacques et al., 2015). These patterns are generally characteristic of particular specificity subgroups. Thus, all CDPSs predicted to mainly synthesize cAA originate from Legionellaceae and contain several insertions and an additional C-terminal domain of unknown function.

## Discussion

Many natural products are synthesized around a 2,5-diketopiperazine core. They are of considerable interest due to the great diversity of their biological activities associated with the various chemical modifications that can occur occur on the initial cyclodipeptide scaffold (Borthwick, 2012). Although numerous DKPs have been identified in bacteria, most have been isolated and characterized from fungi of the *Aspergillus* and *Penicillium* species. The number of characterized biosynthetic pathways for DKPs is relatively low relative to their known diversity (Belin et al., 2012; Giessen and Marahiel, 2015). Ten pathways that involve NRPSs have been decoded (Belin et al., 2012; Giessen and Marahiel, 2015), whereas only six have been shown to depend on CDPSs: albonoursin (Lautru et al., 2002), pulcherrimin (Cryle et al., 2010), mycocyclosin (Belin et al., 2009), methylated ditryptophan (Giessen et al., 2013a), nocazines (Giessen et al., 2013b), and, most recently bicyclomycin (Meng et al., 2017; Patteson et al., 2017). In this context, mapping the diversity of cyclodipeptides attainable by CDPSs allows the identification of new gene clusters responsible for the synthesis of DKPs with high pharmacological potential, as illustrated by the recent identification of the biosynthetic pathway of bicyclomycin, which involves a CDPS predicted to belong to the cLI/cLL-synthesizing group (Patteson et al., 2017).

CDPSs are often promiscuous enzymes. Our early works on CDPSs led us to propose that CDPSs generally produce one major cyclodipeptide and several minor cyclodipeptides according to the general formula cyclo(AA_1_-X), in which AA_1_ is the preferred amino acyl accommodated in P1 (i.e., the amino acyl common to the most highly produced cyclodipeptides) and X is a variable amino acyl accommodated in P2 (Moutiez et al., 2017). This proposition was essentially based on results obtained for AlbC. Indeed, its preferred amino acyl is phenylalanyl, which was shown to be accommodated in P1 (Moutiez et al., 2014b). AlbC produces mainly cFL, along with up to 12 other cyclodipeptides, four of which contain phenylalanine and are produced in significant quantities (Gondry et al., 2009). These pioneering studies showed that the preferred amino acyl is accommodated in P1 with high specificity and suggested lower specificity for binding of the second aa-tRNA. However, characterization of the CDPS of *A. mirum*, which exclusively synthesizes cWW suggests that the binding of both aa-tRNAs can be stringent (Giessen et al., 2013a; Jacques et al., 2015). These results suggest that the amino acid preferentially used by a CDPS can be accommodated by either the P1 or P2 pockets. The CDPS from *N. prasina* was characterized by Brockmeyer and Li (2017) during the writing of this manuscript. According to our model, this enzyme belongs to the cFX-synthesizing group (Supplemental Data Set 4). They found that it synthesizes cFY as the main product (87%) in agreement with our prediction, and minor tyrosyl-containing cyclodipeptides, suggesting a strong determinant of specificity toward the second substrate used by the enzyme. We observed the same type of results for CDPS 66, which also produces mainly cFY (94%) and cYY (6%). This clearly shows that experimental data are needed to identify all cyclodipeptides that can be synthesized by a yet-to-be-characterized CDPS and to possibly deduce a preferred aminoacyl. This also confirms that our predictive approach can only be used to determine the main cyclodipeptide produced, and should not be used to infer the type of cyclodipeptides made by a CDPS.

Analysis of the variability of binding-site residues within a specificity-based group and between phylogenetically close groups provides evidence for specificity-conferring key positions in sequence motifs of the pockets. Comparison of the P1 sequence motif of CDPSs related to CDPS 87, which produces cGE, with those found for the cLE- and cAE-synthesizing group appears to confirm the predominant role of the second residue in the recognition of the first amino acid used by these CDPSs. Previous analyses suggested that the presence of alanine or leucine at this position correlates with the synthesis of cLE or cAE, respectively (Jacques et al., 2015). CDPS 87 has phenylalanine at this position. Its size would prevent the binding of any side chain and favor the presence of a glycyl residue. Such a pattern involving a predominant key residue is also involved in the discrimination between phenylalanyl and tyrosyl moieties in P1 for cFX- and cYY- synthesizing NYH groups. Previous studies carried out on AlbC and Rv2275 identified the presence of an asparagine at position 8 of P1 (L200 in AlbC and N251 in Rv2275) to be essential for the recognition of the tyrosyl (Vetting et al., 2010; Sauguet et al., 2011). Among the newly characterized enzymes, CDPS 67 has a P1 motif closely related to that of the cYY-synthesizing group, with a valine instead of asparagine at position 8 and was shown to produce mainly cFY. The cFX- and cW_2_X-synthesizing groups present a further example. The sequence motifs determined for P1 in both groups are very similar (Table 2 and Table S1). The specific recognition of the tryptophanyl to the detriment of phenylalanyl moiety appears to be related to the presence of the couple (V/I)A at positions 3 and 4 of P1.

This study reveals that different pocket motifs can lead to the preferential recognition of the same amino-acid residue. This had already been noted for CDPSs with similar activities, but belonging to the different subfamilies, NYH and XYP (Jacques et al., 2015); this can be extended to enzymes within the same subfamily. Thus, two different consensus sequences have been identified for the binding of tryptophanyl in P1 pockets of NYH CDPSs. Similarly, different P1 motifs recognize leucyl in XYP CDPSs (e.g., previously characterized CDPS 21 synthesizing cLL vs. cLE- or cLI/cLL synthesizing CDPSs). The specificity code linking the composition of substrate binding pockets to specificity appears to be degenerate and there are multiple strategies to bind the same substrate as for NRPSs (Stachelhaus et al., 1999).

Most of the newly predicted specificity-based groups belong to the NYH subfamily and the prediction only concerns the amino acid recognized by P1 for most. We failed to identify a characteristic pattern for P2 associated with the recognition of a specific residue, except for the XYP CDPSs synthesizing cXE cyclodipeptides. Previous studies on AlbC indicated that most of the recognition of the first substrate is centered on the aminoacyl moiety of the aa-tRNA substrate, whereas recognition of the second substrate involves both the aminoacyl and the tRNA moieties (Moutiez et al., 2014b). The tRNA binding sites are still unknown. Their identification, and particularly the identification of the binding site for the tRNA moiety of the second substrate, would be a decisive step toward improving the predictive model for the second amino acid incorporated by CDPSs. The results obtained for the three CDPSs (63, 64, and 70) that synthesize Trp-containing cyclodipeptides with cWW as the main product are particularly indicative of the importance of the tRNA sequence for the specific recognition of the second substrates. The previously characterized group of cWW-synthesizing enzymes exclusively produced this cyclodipeptide, also showing strong specificity toward the second amino acid (Giessen et al., 2013a; Jacques et al., 2015). Both CDPSs 63 and 10 can synthesize approximately 10 cyclodipeptides other than cWW, some in significant quantities. The chemical and physical features of the second incorporated amino acid are surprisingly diverse, ranging from large hydrophobic residues, such as Trp or Leu, to small ones, such as Ala or Pro, and even polar ones, such as Ser or Glu/Gln. This suggests that specificity determinants toward the amino acid moiety are particularly weak. The identity of the N^1^-N^72^ base pair of the tRNA moiety was found to be the major determinant of specificity for the recognition of the second substrate by AlbC. In the case of CDPSs 63 and 70, specific interactions between tRNA bases and the CDPS probably occur at positions other than the first base pair, as the composition of this pair largely differs between tRNA moieties of all substrates used by these enzymes.

The proportion of CDPSs with predictable activity is significantly higher for those of the NYH than XYP subfamily. It is important to keep in mind that all structural data concerning the identification of P1 and P2 were obtained with NYH CDPSs. Although the predictive model was relevant for several XYP groups, we cannot exclude that the positions of the residues lining the pockets are not strictly conserved between XYP and NYH CDPSs, nor throughout each subfamily. Determination of the tridimensional structures of XYP enzymes will probably refine the definition of the two binding pockets. Parallels can be drawn with the evolution of the predictive models of amino acid recognition by adenylation domains of NRPSs. The first models were based on the identification of amino acids lining the binding pocket of the aminoacyl moiety (Stachelhaus et al., 1999; Challis et al., 2000) and were further refined to include all active site residues close to the substrate (Rausch et al., 2005). Such an approach makes it possible to account for potential differences in the size and geometry of the active site. This is probably the future of CDPS activity prediction, as structural knowledge on CDPSs will continue to increase.

The second important aspect of this study is the expanded number of identified cyclodipeptides synthesized by CDPSs and, as a consequence, the expanded diversity of additional complex DKPs potentially attainable through CDPS-dependent pathways. Among the new cyclodipeptides identified, there were many tryptophanyl-containing cyclodipeptides produced in large quantities as the main products of several CDPSs. Until now, cWW was the only tryptophanyl-containing cyclodipeptide known to be synthesized by CDPSs in significant amounts (Seguin et al., 2011; Giessen et al., 2013a; Jacques et al., 2015). Tryptophanyl-containing cyclodipeptides constitute the largest source of precursors for DKPs with therapeutic potential (Li, 2010; Borthwick, 2012). The intrinsic chemical reactivity of the tryptophan side chain makes it the center of a wide variety of reactions. Thus, electrophilic substitution reactions can occur at all positions of its indole ring and involve modifications as varied as methylation, hydroxylation, nitration, and prenylation, among others (Alkhalaf and Ryan, 2015). Of special interest is the cyclo (L-Trp-L-Pro) or brevianamide F, that was found to be the most prevalent precursor of valuable DKPs, such as brevianamides, tryprostatins, norgeamides, and fumitremorgins (Borthwick, 2012; Gu et al., 2013). The biosynthetic pathway of brevianamide F was characterized in *Aspergillus fumigatus* and shown to depend on an NRPS (Maiya et al., 2006). Both CDPSs 74 (*S*. sp. NRRL S-1868) and 75 (*S*. sp. NRRL F-5123) synthesize substantial amounts of cWP, demonstrating that there is a bacterial CDPS-dependent alternative for the synthesis of brevianamide F. The surrounding genes of these CDPSs encode numerous putative tailoring enzymes, such as cytochrome P450 and reductases/oxidases in the case of CDPS 74 (NCBI entry NZ_JOGD01000018.1) and, among others, several putative methyltransferases for CDPS 75 (NCBI entry NZ_JOHY01000010.1). This suggests the existence of larger gene clusters that may be responsible for the synthesis of modified cyclo(L-Trp-L-Pro) in these *Streptomyces* species. Their characterization may lead to the identification of new pathways for already identified DKPs or the identification of new derivatives.

Several newly characterized CDPSs produce other tryptophanyl-containing cyclodipeptides that could also be potential precursors of more complex DKPs. CDPSs 69 and 71 synthesize cWY, for which the scaffold can be identified in some thaxtomin derivatives (King and Lawrence, 1996; King and Calhoun, 2009; Borthwick, 2012; Giessen and Marahiel, 2014). CDPS 68 synthesizes cWL, which is related to cyclomarazines (Schultz et al., 2008). The already identified biosynthetic pathways for these DKPs involve NRPSs. The CDPSs characterized herein are all part of larger putative gene clusters that include putative cytochrome P450, methyltransferases, and other tailoring enzymes. The identification of CDPS-dependent pathways for such molecules, i.e., small efficient biocatalysts compared to NRPSs, may facilitate the development of new synthetic pathways to produce novel active DKPs.

In conclusion, our work expands the first predictive model that we proposed for the specificity of CDPSs (Jacques et al., 2015). The identification of CDPS products constitutes the first step toward the deciphering of CDPS-dependent pathways. The identification of CDPS-synthesized cyclodipeptide precursors of high-value DKPs makes CDPSs highly attractive as efficient systems for biological synthetic approaches.

## Author contributions

MG and J-LP obtained funding. MM performed bioinformatics analyses. IJ and MB performed cloning experiments and prepared culture supernatants. RT performed LC/MS/MS analyses. MM and NC analyzed MS/MS data. MM, MG, PB, J-LP, and JS analyzed and discussed the results. MM and MG wrote the manuscript. All authors participated in the production of the final version of the manuscript.

## Author note

During the editing proccess of this paper, Skinnider et al. published an algorithm to identify CDPSs and predict their aminoacyl-tRNA substrates (Skinnider et al., 2018). We looked at the cyclodipeptides they predicted (Additional File 4) for the 32 CDPSs we experimentally characterized. The prediction is accurate for 5 of 32 enzymes (16.1%).

### Conflict of interest statement

The authors declare that the research was conducted in the absence of any commercial or financial relationships that could be construed as a potential conflict of interest.

## Supplementary material

Figure S1 Current diversity of the cyclodipeptides produced by the NYH and XYP CDPS subfamilies. The amino acids forming the cyclodipeptide ring are indicated in the one-letter code at the top and right. Previously identified cyclodipeptides are in blue, cyclodipeptides identified with the present set of CDPSs are in orange. Light colors indicate cyclodipeptides detected in low amounts (UV peak area at 214 nm less than 200). **(A)** Cyclodipeptides produced by NYH CDPSs. **(B)** Cyclodipeptides produced by XYP CDPSs.

Table S1 Related to Materials and Methods: Related ion masses m/z used for identification of cyclodipeptides.

Table S2 Related to Table 3: Consensus sequences for the specificity groups identified.

Supplemental Data Set 1 Database information and sequence data relative to characterized CDPSs.

Supplemental Data Set 2 MS2 and MS3 spectra of cyclodipeptides (standards and newly identified).

Supplemental Data Set 3 LC-MSMS analyses of the supernatants of BL21AI-pREP4 expressing the recombinant CDPSs.

Supplemental Data Set 4 Prediction of CDPS groups having the same cyclodipeptide-synthesizing activities.

Supplemental Data Set 5 Sequence alignment of CDPSs, used to generate the phylogenetic tree.

Supplemental Data Set 6 High-resolution view of the phylogenetic tree of CDPSs, with aLRT values (June 2017).
