# Supplementary material for: A Comprehensive Overview of the Cyclodipeptide Synthase Family Enriched with the Characterization of 32 New Enzymes
Source: Front Microbiol. 2018 Feb 12;9:46. doi: 10.3389/fmicb.2018.00046 (PMC5816076; doi:10.3389/fmicb.2018.00046)
Supplement: Figure S1 — Current diversity of the cyclodipeptides produced by the NYH and XYP CDPS subfamilies. The amino acids forming the cyclodipeptide ring are indicated in the one-letter code at the top and right. Previously identified cyclodipeptides are in blue, cyclodipeptides identified with the present set of CDPSs are in orange. Light colors indicate cyclodipeptides detected in low amounts (UV peak area at 214 nm less than 200). (A) Cyclodipeptides produced by NYH CDPSs. (B) Cyclodipeptides produced by XYP CDPSs. [file Image1.pdf]

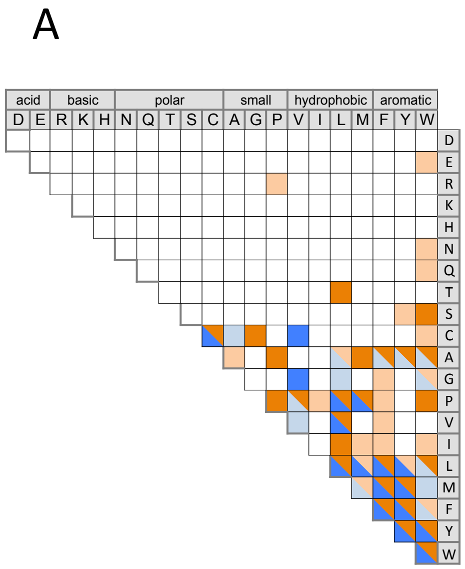

Figure S1. Current diversity of the cyclodipeptides produced by the NYH and XYP CDPS subfamilies. The amino acids forming the cyclodipeptide ring are indicated in the one-letter code at the top and right. Previously identified cyclodipeptides are in blue, cyclodipeptides identified with the present set of CDPSs are in orange. Light colors indicate cyclodipeptides detected in low amounts (UV peak area at 214 nm less than 200). (A) Cyclodipeptides produced by NYH CDPSs. (B) Cyclodipeptides produced by XYP CDPSs.
